# Association between the gut microbiotic composition and dietary patterns in hypertensive elderly patients: a cross-sectional study

**DOI:** 10.1186/s12986-025-00963-8

**Published:** 2025-07-07

**Authors:** Hsi-Cheng Hung, Yuan-Yuan Lin, Wan-Jung Tien, Yu-Yoh Chen, Suh-Ching Yang

**Affiliations:** 1 School of Nutrition and Health Sciences, Taipei Medical University, Taipei, 11031 Taiwan; 2 Department of Family Medicine, Cathay General Hospital, Taipei, 10630 Taiwan; 3 Diet and Nutrition Section, Cathay General Hospital, Taipei, 10630 Taiwan; 4 Graduate Institute of Health and Biotechnology Law, Taipei Medical University, Taipei, 11031 Taiwan; 5 Research Center of Geriatric Nutrition, College of Nutrition, Taipei Medical University, Taipei, 11031 Taiwan; 6 Nutrition Research Center, Taipei Medical University Hospital, Taipei, 11031 Taiwan; 7 School of Gerontology and Long-Term Care, College of Nursing, Taipei Medical University, Taipei, 11031 Taiwan

**Keywords:** Hypertension, Dietary pattern, Nutrient intake, Microbiotic composition, Older adult

## Abstract

**Background:**

Many studies on gut microbiota and hypertension have not focused on detailed dietary intake and eating habits, especially in older adults. This cross-sectional study aimed to examine the gut microbiota profiles of hypertensive elderly individuals in relation to their dietary patterns and nutrient intake.

**Methods:**

Twenty hypertensive patients and 21 age-matched healthy controls (aged 65–80 years) were recruited from Cathay General Hospital (Taipei, Taiwan). Data collected included anthropometric measurements, blood pressure, blood biochemical analyses, and dietary intake (24-h recall and food frequency questionnaires) and fecal microbiotic composition (via 16S rRNA sequencing).

**Results:**

Hypertensive patients had significantly higher BMI, waist circumference, and systolic blood pressure. They also showed lower levels of Bacteroides caccae and *Barnesiella*, and higher levels of *Enterobacteriaceae*, *Enterobacter*, *Acidaminococcus*, and *Bacteroides plebeius*. *Bacteroides caccae* abundance was negatively correlated with the intake of saturated fats, sodium, staple foods (e.g., bread, steamed buns, noodles), nut oils, and seasonings.

**Conclusions:**

Hypertensive patients showed distinct gut microbiota profiles, with lower levels of *Bacteroides caccae* and *Barnesiella*, and higher levels of Enterobacteriaceae-related taxa. The abundance of *Bacteroides caccae* was negatively associated with the intake of saturated fats, sodium, and staple foods, suggesting a link between diet, gut microbiota, and hypertension.

## Introduction

Hypertension is one of the most common chronic diseases worldwide and a major risk factor for cardiovascular, brain, and kidney diseases. According to the latest data from the World Health Organization (WHO), approximately 1.3 billion people aged 30–79 years worldwide had hypertension in 2023 [1]. In Taiwan, based on the results of the 2017–2020 National Nutrition and Health Survey, the prevalence of hypertension among individuals aged 18 years and older had reached 26.8%, and the prevalence increases with age [2]. Risk factors for hypertension include an unhealthy diet (such as excessive salt consumption, high intake of saturated fats and trans fats, and low consumption of fruits and vegetables), physical inactivity, tobacco and alcohol consumption, being overweight or obese, being over 65 years of age, and having coexisting conditions such as diabetes or kidney disease [3]. On the other hand, the elderly in Asia are increasingly vulnerable due to the “triple burden” of an aging population, hypertension, and mental health issues [4]. Therefore, the causes of hypertension in the elderly and its impacts on health are crucial health issues for growing aged populations in Asia. Although antihypertensive treatments are widely implemented, a 2021 community-based survey in Taiwan revealed that over 40% of patients with hypertension still failed to achieve optimal blood pressure control, particularly among older adults [5]. This highlights the urgent need for novel and complementary strategies to regulate blood pressure beyond conventional pharmacological approaches. Among these, the modulation of gut microbiota has emerged as a promising and innovative direction.

Animal models have demonstrated gut microbial effects on blood pressure (BP) [6–8]. Hypertension was induced in normotensive rats through transplantation of cecal contents from hypertensive rats [6, 9]. The gut microbiota generates various metabolites, including trimethylamine-N-oxide (TMAO), short-chain fatty acids (SCFAs), corticosterone, hydrogen sulfide (H_2_S), choline, bile acids (BAs), indole sulfate, and lipopolysaccharides (LPSs). Among these, SCFAs, TMAO, BAs, H_2_S, and LPSs are closely linked to the development of hypertension [10–13]. The CARDIA and HELIUS studies, two prospective cohort studies conducted in the United States and Europe with 529 and 4672 participants, respectively, both found that hypertensive patients exhibited significantly lower α and β diversities in their gut microbiota compared to individuals with normal BP [14, 15]. Additionally, there were significant differences in the abundances of certain bacterial species between the two groups, although most of the bacterial species identified in the two studies did not overlap. A study conducted in various regions of China found that gut microbiotic compositions varied across different regions and ethnic groups, indicating that lifestyle and ethnicity might influence the gut microbiotic composition [16].

While changes in the gut microbiotic composition have been linked to hypertension, most such studies focused on Western populations, which might not be applicable to Asian countries including Taiwan, a region with distinct ethnicities and dietary habits. Therefore, in this study, we investigated relationships among dietary patterns, the gut microbiota, and hypertension in elderly individuals, using data from Taiwan to explore how regional dietary habits and microbiotic composition influence BP regulation.

## Materials and methods

### Study design and participant recruitment

This study was approved by the Institutional Review Board (IRB) of Cathay General Hospital (Taipei, Taiwan), Cathay Medical Foundation (CGH-P110063, approved on December 24, 2022). The international registration number is NCT05057039, and it was listed on ClinicalTrials.gov PRS and reviewed on April 14, 2023. Participants were recruited from elderly individuals undergoing health check-ups at Cathay General Hospital. The inclusion criteria for the hypertension group were individuals aged 65–80 years who had a systolic BP exceeding 140 mmHg or a diastolic BP exceeding 90 mmHg during a health check-up, or those who had taken antihypertensive medication within the past month. For the normotensive group, participants were age- and sex-matched individuals who had a systolic BP below 139 mmHg, a diastolic BP below 89 mmHg, and had not taken antihypertensive medication within the past month. Exclusion criteria included individuals over 80 years old, and those who had used antibiotics in the past month, consumed probiotics in the past week, had a history of inflammatory bowel disease or gastrointestinal surgery, suffered from chronic gastrointestinal diseases requiring long-term medication, had experienced acute gastrointestinal symptoms such as vomiting or diarrhea in the past week, had been diagnosed with secondary hypertension, or were unable to complete the dietary assessment questionnaire. Participants who were willing to participate and met the eligibility criteria provided informed consent before data collection. The collected data included height, weight, body-mass index (BMI), BP, pulse, medical history, and blood and biochemical test results. Additionally, a registered dietitian conducted dietary records, food frequency questionnaires, and nutritional analyses. Participants were also provided with a fecal sample collection kit to obtain stool samples for a gut microbiotic analysis.

### Basic information, anthropometric data, and BP

For anthropometric measurements, researchers used a fully automated height and weight scale (HW-210, Universal Weight Electronics, New Taipei City, Taiwan) at the Cathay General Hospital Health Examination Center to measure participants’ height and weight and calculate their BMI.

BP was measured using office BP monitoring with a fully automated medical-grade electronic sphygmomanometer (HBP-9030, Omron, Kyoto, Japan). Before the measurement, participants were required to rest for at least 5 min. A nurse assisted in the measurement process, recording three BP and pulse readings, with the average value used for the statistical analysis.

### Blood biochemical analysis

Blood biochemical data were analyzed using a fully automated hematology analyzer (XN-10/XN-20, Sysmex, Kobe, Japan) to measure hemoglobin, the white blood cell (WBC) count, and platelet count. Additionally, biochemical analyses were conducted using a fully automated analyzer (AU5800, Beckman Coulter, Brea, CA, USA). The analyzed parameters included liver function markers (aspartate aminotransferase, AST; and alanine aminotransferase, ALT), kidney function marker (creatinine), and metabolism-related indicators such as fasting blood glucose, total cholesterol (TC), triglycerides (TGs), high-density lipoprotein cholesterol (HDL-C), low-density lipoprotein cholesterol (LDL-C), uric acid, albumin, and globulin levels.

### Dietary intake

On the day of enrollment, participants underwent an interview with a registered dietitian to complete a 24-h dietary recall and a food frequency questionnaire (FFQ). The 2016 Edition of the Simplified Nutrition Calculation Table (Microsoft Excel) was used to assess participants’ total energy, macronutrient, dietary fiber, vitamin, and mineral intake levels. Food intake was categorized based on six major food groups, with further classification of staple foods into whole grains, legumes, tubers, and others (e.g., bread, steamed buns, noodles). Additional analyses were conducted on seasoning usage, cooking methods, processed meats, and pickled foods to evaluate their potential impacts on BP.

The calculation method for dietary frequency was based on a previous study, by converting weekly and monthly intake frequencies into daily equivalents [17]. For instance, food consumed one or two times per week was averaged to 1.5 times per week, corresponding to 0.21 times per day, while once per month was converted to 0.03 times per day.

### Fecal microbiotic analysis

This study used a 16S ribosomal (r)RNA variable region analysis to classify the fecal microbiota. Fecal samples were stored at -20 °C until being analyzed. DNA was extracted from fecal samples using a QIAamp Fast DNA Stool Mini Kit (Qiagen, Hilden, Germany). The V3 + V4 region was amplified by a polymerase chain reaction (PCR) for the microbiotic analysis. The first round of PCR amplification was carried out using Kapa HiFi HotStart ReadyMix (KapaBiosystems, Wilmington, MA, USA) with 0.2 µM forward and reverse primers targeting the V3 + V4 region, and DNA was purified using Agencourt AMPure XP Reagent beads (Beckman Coulter, Brea, CA, USA). In the second round of the PCR, Nextera XT Index 1 and 2 (Illumina, San Diego, CA, USA) were added, and a quantitative (q)PCR was performed. Samples were then uniformly mixed and sequenced using the Illumina MiSeq NGS system. After sequencing, more than 80,000 paired-end 300-bp sequences were generated. These sequences were classified and analyzed using the DADA2 workflow and SILVA v132 taxonomic database.

### Statistical analysis

SPSS statistical software (SPSS vers. 19, IBM, Chicago, IL, USA) was used for data analysis. For categorical variables, data are presented as counts (*n*) and percentages (%); for continuous variables, the Kolmogorov-Smirnov test was first applied to assess the normality of the distribution. If variables followed a normal distribution, the data were expressed as means and standard deviations (SDs); if variables did not follow a normal distribution, data are presented as medians and interquartile ranges (IQRs). To compare differences between groups, the Chi-squared test was used for categorical variables. For continuous variables, an independent *t*-test was used for normally distributed variables, and the Mann-Whitney *U*-test was used for non-normally distributed variables. Furthermore, to explore correlations between the gut microbiotic composition and clinical factors or dietary frequency, Spearman’s rank correlation coefficient analysis was used. A *p* value of < 0.05 was set as the threshold for statistical significance.

In the fecal microbiotic analysis, this study used the MicrobiomeAnalyst platform to perform richness and diversity analyses, as well as differential abundance analyses, comparing the hypertensive group and healthy control group [18, 19]. The α diversity of the gut microbiota was assessed using five indices: Observed, Chao1, ACE (abundance-based coverage estimator), Shannon index, and Simpson index. β diversity was visualized using a principal coordinates analysis (PCoA), with diversity differences between groups calculated using three methods: Bray-Curtis distance, weighted UniFrac, and unweighted UniFrac. To assess the statistical significance of differences between groups, a permutational multivariate analysis of variance (PERMANOVA) was applied. Additionally, for the differential abundance analysis of the fecal microbiota, this study used the edgeR statistical method for single-factor statistical comparisons, and applied the Benjamini-Hochberg method to adjust original *p* values for the false discovery rate (FDR), yielding corresponding q values. Finally, a q value of < 0.05 was set as the threshold for statistical significance to determine whether there were significant differences in gut microbiotic compositions between the hypertensive group and normotensive group.

## Results

### Basic information and characteristics

**Fig. 1:**
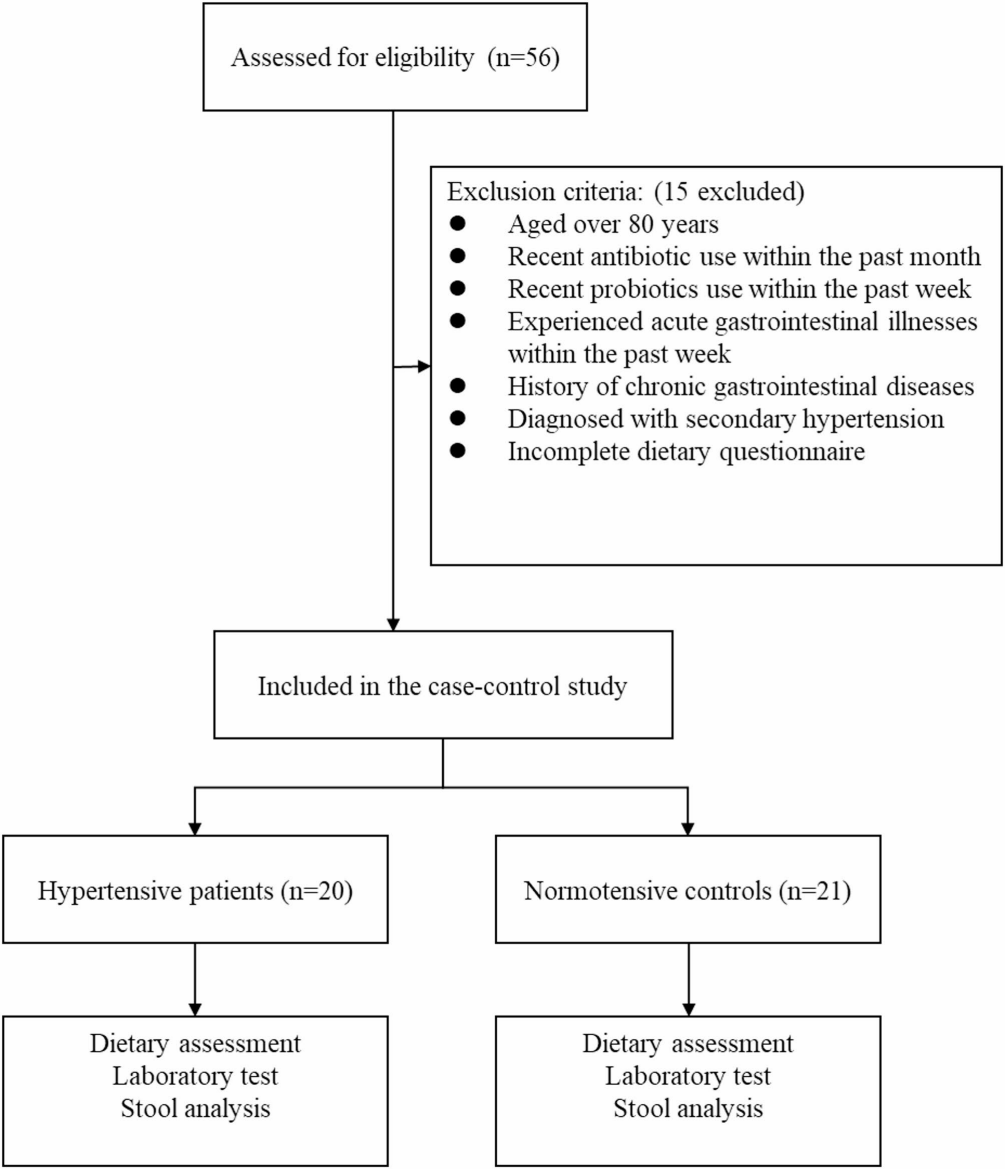
Participant enrollment and flowchart

Initially 56 participants were enrolled in this study, among whom 15 were excluded due to failing to meet the inclusion criteria. Ultimately, 20 hypertensive patients and 21 normotensive individuals were included (Fig. 1). When comparing the characteristics of the two groups, there were no significant differences in terms of gender, age, diabetes, or hyperlipidemia. However, compared to the normotensive group, the hypertensive group showed a significantly higher BMI, waist circumference, and systolic BP (Table 1).

**Table 1:** Characteristics of hypertensive patients and healthy controls

Variable	Hypertensive patients (*N* = 20)	Normotensive controls (*N* = 21)	*p* value
Male (%)	10 (50)	10 (48)	0.883
Age (years)	72 ± 5.3	70 ± 3.6	0.086
Body-mass index (kg/m^2^)	24.8 ± 3.4	21.2 ± 2.5	< 0.001
Waist circumference (cm)	83.4 ± 9.0	76.5 ± 8.9	0.018
Systolic blood pressure (mmHg)	143 ± 14	122 ± 12	< 0.001
Diastolic blood pressure (mmHg)	75 ± 12	70 ± 7	0.166
Diabetes (%)	4 (20)	3 (14)	0.637
Hyperlipidemia (%)	3 (15)	5 (24)	0.489

Values are expressed as the number (%) of patients or the mean ± standard deviation

### Blood biochemical parameters

As shown in Table 2, the TC level was significantly lower in the hypertensive group compared to the normotensive group. However, there were no significant differences between the two groups in other blood-related indicators, including hemoglobin, WBCs, platelets, AST, ALT, creatinine, fasting blood glucose, TGs, HDL-C, LDL-C, uric acid, albumin, and globulin (Table 2).

**Table 2:** Blood analysis of hypertensive patients and healthy controls

Variable	Hypertensive patients (*N* = 26)	Normotensive controls (*N* = 27)	*p* value
Hemoglobin (g/dL)	13.9 ± 1.5	14.5 ± 1.2	0.166
WBCs (1000/µL)	5.4 ± 1.8	5.2 ± 1.2	0.692
Platelets (1000/µL)	224 ± 43	230 ± 64	0.703
Hematocrit (%)	41.4 ± 4.2	42.4 ± 3.2	0.368
RBCs (10^6^/µL)	4.6 ± 0.4	4.7 ± 0.5	0.793
RDW (%)	13.1 ± 1.4	12.7 ± 0.5	0.211
MCV (fL)	89.2 ± 4.2	91.1 ± 4.2	0.156
AST (U/L)	20 ± 5	24 ± 12	0.223
ALT (U/L)	15 ± 8	18 ± 18	0.440
Creatinine (mg/dL)	0.88 ± 0.18	0.84 ± 0.17	0.388
Fasting glucose (mg/dL)	104 ± 15	106 ± 43	0.821
Total cholesterol (mg/dL)	171 ± 40	206 ± 32	0.004
Triglycerides (mg/dL)	95 ± 36	121 ± 48	0.061
HDL-C (mg/dL)	65 ± 13	68 ± 16	0.514
LDL-C (mg/dL)	95 ± 32	120 ± 28	0.015
Uric acid (mg/dL)	5.9 ± 1.2	5.7 ± 0.9	0.584
Albumin (g/dL)	4.4 ± 0.3	4.4 ± 0.2	0.632
Globulin (g/dL)	3.1 ± 0.2	3.1 ± 0.3	0.543

Values are expressed as the number (%) of patients or the mean ± standard deviation

WBCs, white blood cells; RBCs, red blood cells; RDW, red blood cell distribution width; MCV, mean corpuscular volume; AST, aspartate transaminase; ALT, alanine transaminase; HDL-C, high-density-lipoprotein cholesterol; LDL-C, low-density-lipoprotein cholesterol

### Dietary intake

Based on the 24-h dietary recall, there were no significant differences between the hypertensive group and normotensive group in daily intake of calories, macronutrients, vitamins, and minerals (Table 3). Additionally, comparisons of the intake frequencies for the six major food groups, condiments, and cooking methods based on the dietary frequency questionnaire also showed no significant differences between the two groups (Table 4).

**Table 3:** Nutrient intake levels in hypertensive patients and healthy controls

Nutrient	Hypertensive patients (*N* = 20)	Normotensive controls (*N* = 21)	*p* value
Energy (kcal)	1451.6 ± 372.8	1545.3 ± 282.3	0.368
Carbohydrates (% of total kcal)	46.6 ± 4.7	49.6 ± 10.3	0.253
Protein (% of total kcal)	17.1 ± 3.2	17.1 ± 3.5	0.975
Fat (% of total kcal)	36.3 ± 5.2	33.4 ± 9.4	0.231
Saturated fat (g)	16.9 ± 5.1	17.1 ± 6.9	0.942
Trans fat (mg)	165.7 ± 118.2	155.3 ± 115.5	0.779
Cholesterol (mg)	298.7 ± 181.5	253.5 ± 141.9	0.379
Dietary fiber (g)	16.4 ± 5.6	17.6 ± 7.1	0.548
Vitamin A (IU)	16933.7 ± 8821.3	17055.7 ± 6455.1	0.960
Vitamin D (µg)	0.9 ± 0.9	0.8 ± 1	0.772
Vitamin E (mg)	20.6 ± 8.6	22.3 ± 17.7	0.689
Vitamin K_1_ (µg)	1.2 ± 3.5	7 ± 21.4	0.247
Vitamin B_1_ (mg)	0.9 ± 0.4	1 ± 0.3	0.382
Vitamin B_2_ (mg)	1.1 ± 0.5	1 ± 0.4	0.783
Niacin (mg)	12.5 ± 6.2	15.3 ± 5.9	0.140
Vitamin B_6_ (mg)	1.4 ± 0.6	1.4 ± 0.5	0.900
Vitamin B_12_ (µg)	3.5 ± 3.8	3.3 ± 2.6	0.867
Folate (µg)	293.9 ± 83.7	301.9 ± 103.6	0.788
Vitamin C (mg)	168.4 ± 82.4	164.2 ± 67.5	0.861
Sodium (mg)	916.2 ± 411.2	785.1 ± 361.7	0.284
Potassium (mg)	2148 ± 584.3	2228.6 ± 613.4	0.669
Calcium (mg)	726.5 ± 261.5	765.6 ± 369.4	0.699
Magnesium (mg)	258.6 ± 67.7	296.2 ± 125.9	0.244
Iron (mg)	9.8 ± 2.5	10.4 ± 4	0.548
Zinc (mg)	7.8 ± 2.3	8.4 ± 2.6	0.392
Phosphorus (mg)	905.5 ± 296.4	981.8 ± 318.1	0.432
Copper (mg)	0.2 ± 0.2	0.2 ± 0.1	0.496
Manganese (mg)	0.1 ± 0.3	0.1 ± 0.2	0.765

Values are expressed as the mean ± standard deviation

**Table 4:** Dietary intake frequencies of food groups from a food frequency questionnaires in hypertensive patients and healthy controls

Food group (servings/day)	Hypertensive patients (*N* = 20)		Normotensive controls (*N* = 21)		*p* value
Staple foods (whole grains)	0.750	(0.210, 1.000)	1.000	(0.355, 2.000)	0.133
Staple foods (beans)	0.030	(0.010, 0.030)	0.030	(0.010, 0.120)	0.447
Staple foods (root vegetables)	0.030	(0.010, 0.428)	0.210	(0.030, 0.210)	0.555
Staple foods (others)	2.000	(1.000, 2.000)	1.000	(1.000, 2.000)	0.215
Dairy and dairy products	0.120	(0.030, 1.000)	0.210	(0.030, 1.000)	0.692
High-fat meats	0.420	(0.060, 1.770)	1.000	(0.240, 2.000)	0.326
Medium-fat meats	1.000	(0.566, 2.000)	1.000	(0.420, 2.000)	0.829
Low-fat meats	1.000	(0.296, 1.750)	1.000	(0.420, 2.000)	0.914
Processed meats	0.060	(0.060, 0.420)	0.060	(0.060, 0.710)	0.988
Eggs	0.750	(0.500, 1.000)	1.000	(0.500, 1.000)	0.721
Soybeans and soy products	1.000	(0.566, 2.000)	2.000	(1.000, 4.000)	0.226
High cholesterol foods	0.030	(0.030, 0.030)	0.030	(0.030, 0.030)	0.551
Vegetables	2.000	(2.000, 3.500)	3.500	(2.000, 3.500)	0.319
Fruits	2.000	(1.000, 3.125)	2.000	(1.000, 2.000)	0.978
Oils and nuts/seeds	2.000	(2.000, 3.500)	2.000	(1.000, 3.500)	0.562
Seasonings	2.000	(2.000, 3.500)	2.000	(2.000, 3.500)	0.736
Pastries and snacks	0.120	(0.030, 0.428)	0.210	(0.030, 0.500)	0.695
Pickled foods	0.030	(0.030, 0.165)	0.030	(0.030, 0.210)	0.673
Canned foods	0.030	(0.030, 0.030)	0.030	(0.030, 0.030)	0.323
Instant noodles	0.010	(0.010, 0.030)	0.010	(0.010, 0.030)	0.381
Sugary beverages	0.030	(0.030, 0.030)	0.030	(0.030, 0.030)	0.583
Alcoholic beverages	0.010	(0.010, 0.025)	0.010	(0.010, 0.120)	0.663
Cooking method - stir-fry	1.000	(0.625, 1.000)	1.000	(0.210, 2.000)	0.553
Cooking method - deep-fry	0.010	(0.010, 0.030)	0.010	(0.010, 0.020)	0.814
Cooking method - boil/blanch	1.000	(0.500, 1.000)	1.000	(0.355, 2.000)	0.978
Cooking method - steam	0.210	(0.075, 0.875)	0.210	(0.030, 1.000)	0.924
Cooking method - braise	0.210	(0.030, 0.500)	0.030	(0.010, 0.210)	0.246

Weekly and monthly intake frequencies were converted into daily intake frequencies. Multiplying the frequency and servings of each food group and summing them by category yields the daily intake servings. The Mann-Whitney *U*-test was used between the two groups. Values are expressed as the mean (25th, 75th percentiles)

### Fecal microbiotic composition

**Fig. 2:**
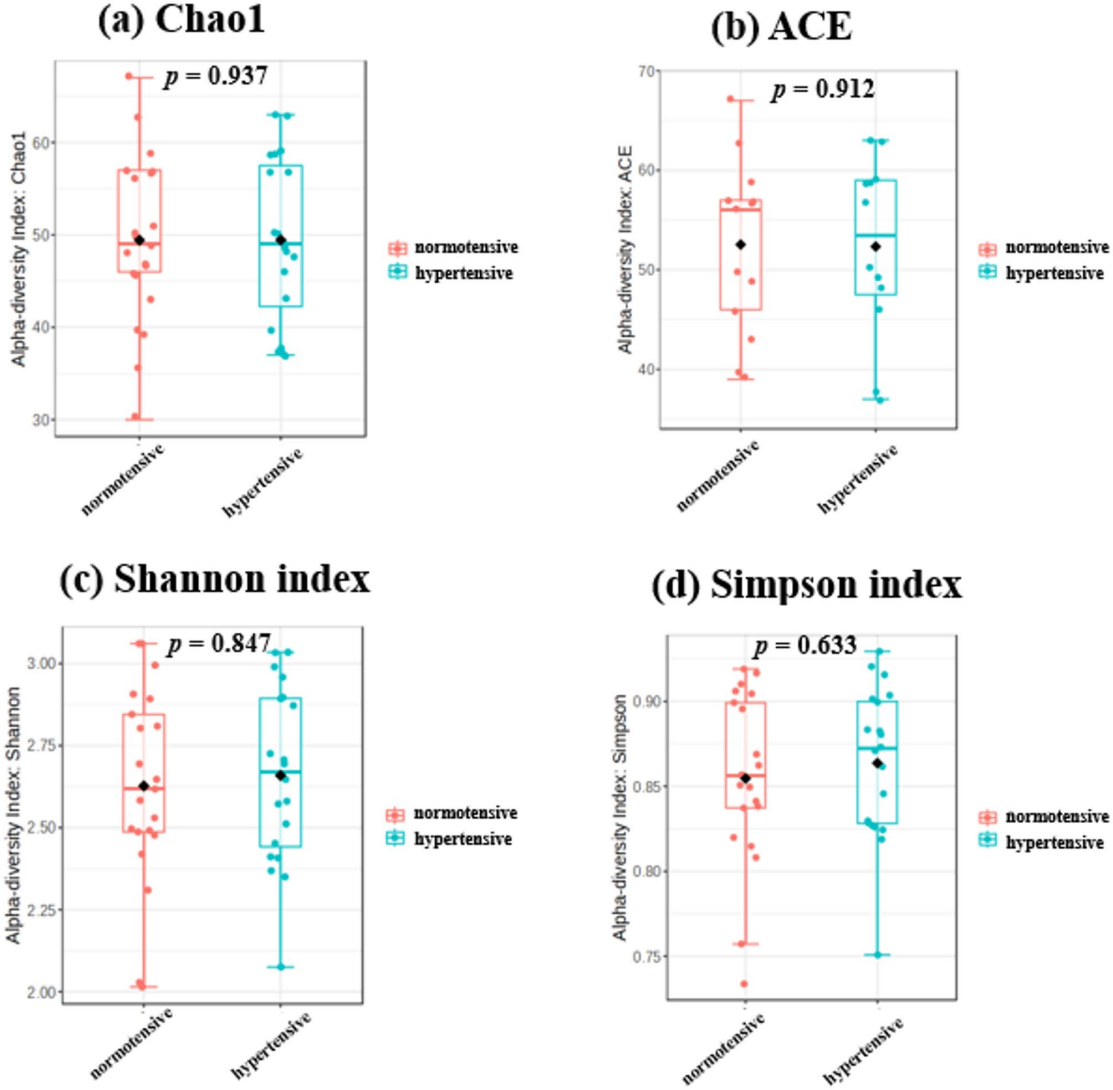
Analysis of gut microbiota α-diversity in hypertensive patients and normotensive individuals. ACE, abundance-based coverage estimator

**Fig. 3:**
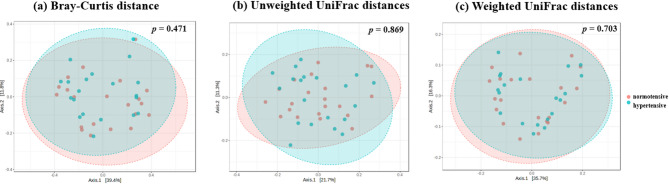
Analysis of gut microbiota β-diversity in hypertensive patients and healthy controls in a principal coordinate analysis (PCoA). (**a**) Bray-Curtis distance, (**b**) unweighted UniFrac distances, (**c**) weighted UniFrac distances

The α diversity analysis showed no significant differences between hypertensive patients and healthy controls in the Observed, Chao1, ACE, Shannon, and Simpson indices, indicating that there were no notable differences in the richness and diversity of the gut microbiota between the two groups (Fig. 2). Similarly, in the β diversity analysis, calculations using Bray-Curtis distance, unweighted UniFrac distance, and weighted UniFrac distance, followed by a PERMANOVA, also revealed no significant differences between the two groups (Fig. 3).

**Fig. 4:**
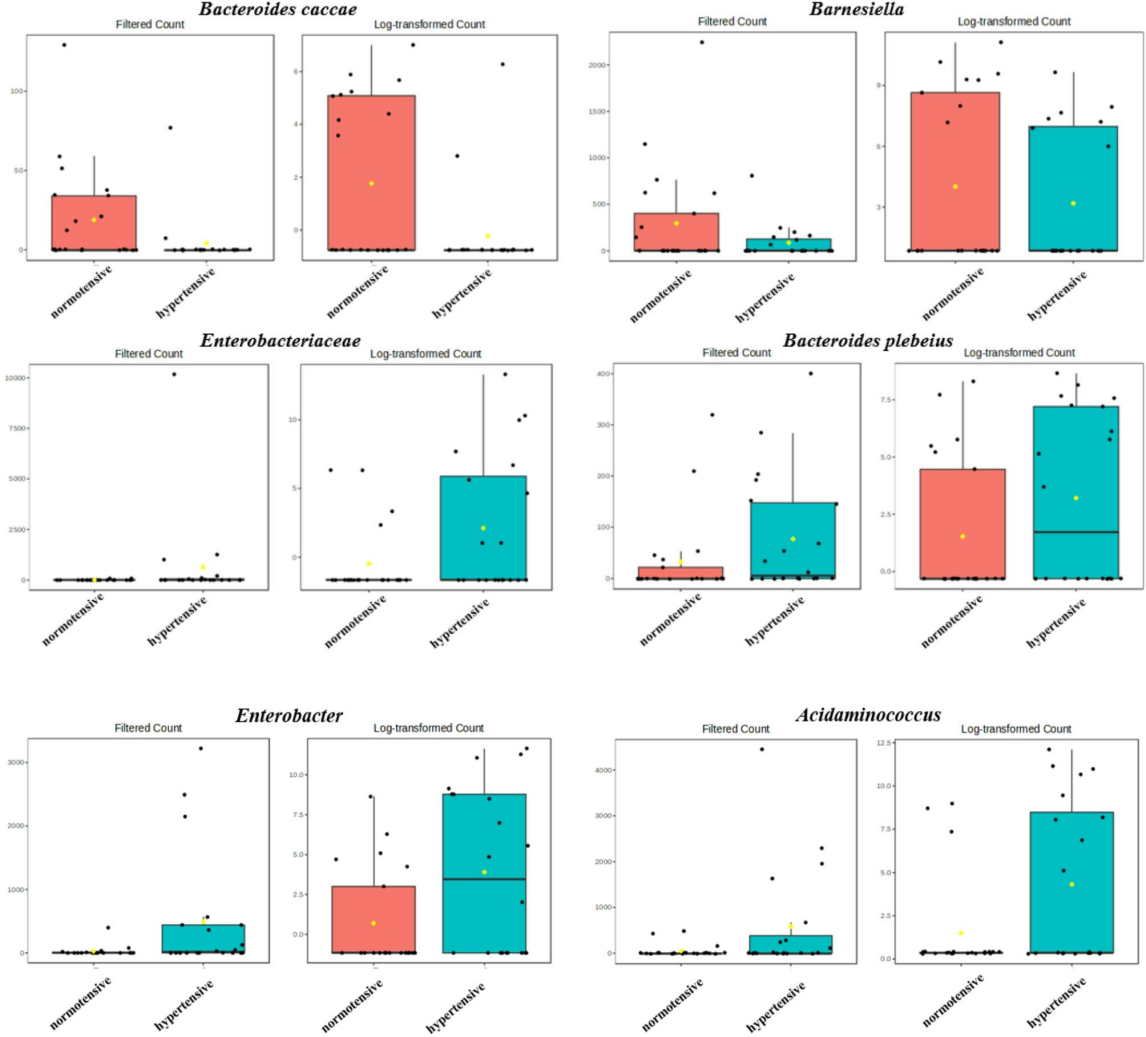
Comparison of the gut microbiota between hypertensive patients and normotensive controls

For gut microbiotic abundances, the study used the edgeR method for statistical analysis and applied the Benjamini-Hochberg method for FDR correction. In total, 153 amplicon sequence variants (ASVs) were compared, and six taxonomic features showed significant differences in relative abundances (Table 5). Among these, *Bacteroides caccae* had a significantly lower relative abundance in hypertensive patients compared to the normotensive group, approximately 2^2.95^ times lower, with a log2 fold change (log2FC) of -2.95. The total sequencing read count of *Bacteroides caccae* accounted for approximately 2^8.41^ counts per million (CPM), with a logCPM of 8.41 (*p* < 0.001, FDR q = 0.013) indicating a significant difference in relative abundances between the two groups. The *Barnesiella* genus also had a significantly lower relative abundance in hypertensive patients compared to the healthy group (FDR q = 0.043). The remaining four taxonomic features showed significantly higher relative abundances in the hypertensive group, including the family Enterobacteriaceae (FDR q = 0.008), *Bacteroides plebeius* (FDR q = 0.043), the *Enterobacter* genus (FDR q = 0.043), and the *Acidaminococcus* genus (FDR q = 0.043) (Fig. 4). A post hoc power analysis was conducted for the six taxa that showed significant differences. Assuming a dispersion value of 0.2 and based on the current sample size (hypertension group, *n* = 20; normotensive group, *n* = 21), as well as the observed effect sizes (log2FC ranging from − 2.95 to 4.71), the estimated statistical power ranged from 0.94 to 0.99.

**Table 5:** Differential abundance analysis of specific taxonomic features between hypertensive patients and healthy controls

Taxonomic feature	log2FC	logCPM	*p* value	FDR q value
*Bacteroides caccae*	-2.95	8.41	< 0.001	0.013
*Barnesiella* genus	-3.17	12.18	0.003	0.043
Enterobacteriaceae family	4.71	12.53	< 0.001	0.008
*Bacteroides plebeius*	2.73	10.41	0.002	0.043
*Enterobacter* genus	3.48	13.44	0.003	0.043
*Acidaminococcus* genus	3.61	13.74	0.003	0.043

The differential abundance analysis was performed using the edgeR statistical method. The Benjamini-Hochberg method was applied to adjust raw *p* values for the false discovery rate (FDR), resulting in *q* values. A *q* value of < 0.05 indicates a significant difference between the two groups. FC, fold change; CPM, counts per million

**Fig. 5:**
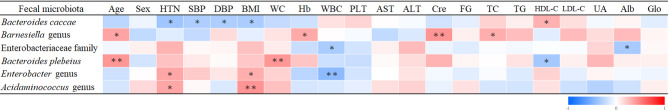
Colored heatmap of correlations between the fecal microbiota and clinical factors. Colored heatmap of Spearman’s rank correlation coefficients for fecal microbiota and clinical factors. The colors refer to the correlation coefficient direction and magnitude, ranging from − 1 (blue) to 1 (red). * *p* < 0.05 (2-tailed). ** *p* < 0.01 (2-tailed). HTN, hypertensive group or control group; SBP, systolic blood pressure; DBP, diastolic blood pressure; BMI, body-mass index; WC, waist circumference; Hb, hemoglobin; WBCs, white blood cells; PLTs, platelets; AST, aspartate transaminase; ALT, alanine transaminase; Cre, Creatinine; FG, fasting glucose; TC, total cholesterol; TGs, triglycerides; HDL-C, high-density-lipoprotein cholesterol; LDL-C, low-density-lipoprotein cholesterol; UA, uric acid; Alb, albumin; Glo, globulin

This study further employed a Spearman’s rank correlation analysis to investigate the relationship between the six gut microbiota species significantly associated with hypertension and various clinical factors and dietary components. Figure 5 presents the correlation analysis results between these six gut bacteria and clinical indicators. In hypertensive or prehypertensive individuals, *Bacteroides caccae* and the *Barnesiella* genus showed negative correlations, whereas the Enterobacteriaceae family, *Bacteroides plebeius*, *Enterobacter* genus, and *Acidaminococcus* genus showed positive correlations. Among these, *Bacteroides caccae*, *Enterobacter*, and *Acidaminococcus* demonstrated statistically significant correlations with hypertension.

As shown in Fig. 5, *Bacteroides caccae* was negatively correlated with the BMI and positively correlated with blood HDL-C levels. The *Barnesiella* genus showed positive correlations with age, hemoglobin, creatinine, and TC levels. The Enterobacteriaceae family was negatively correlated with WBCs and albumin concentrations. Additionally, *Bacteroides plebeius* showed positive correlations with age and waist circumference, but a negative correlation with HDL-C levels. The *Enterobacter* genus was positively correlated with the BMI but negatively correlated with WBC count, while *Acidaminococcus* was positively correlated with the BMI.

**Fig. 6:**
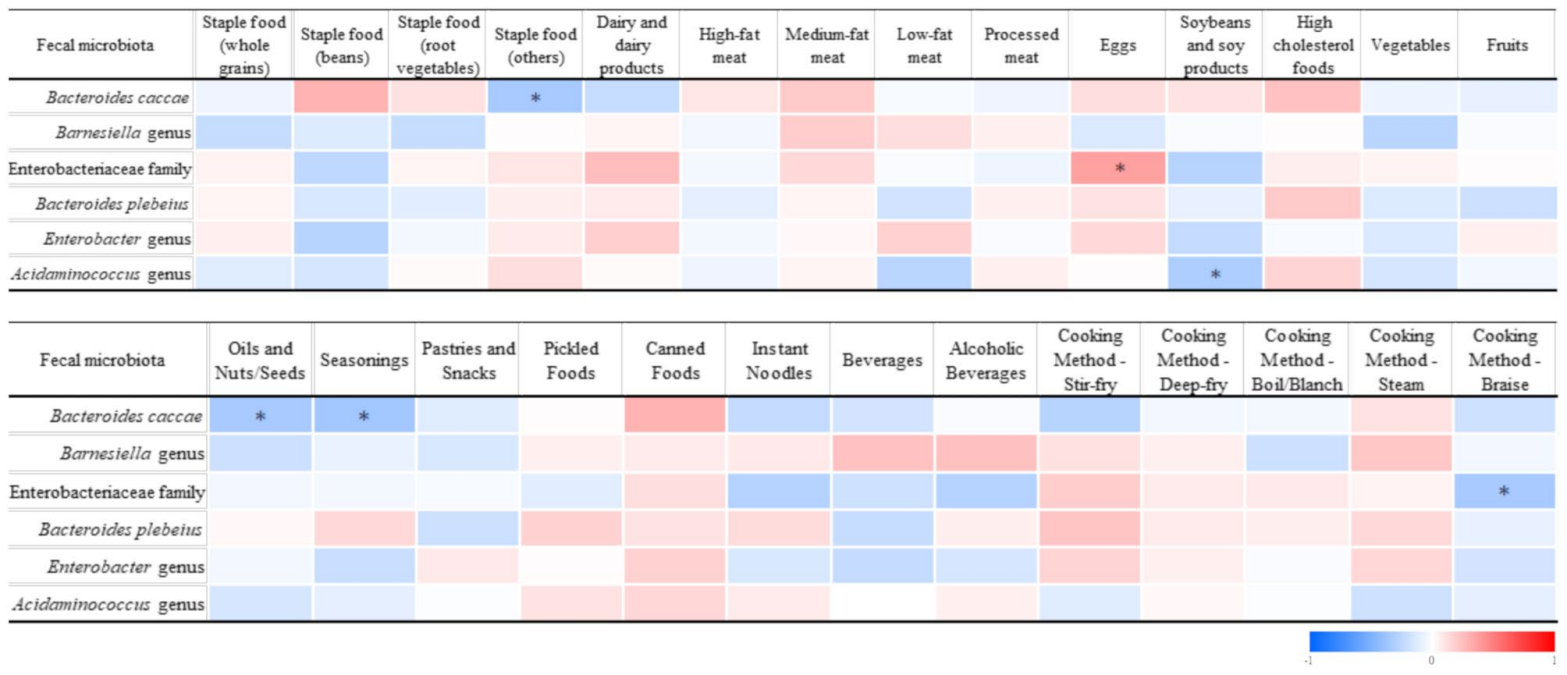
Colored heatmap of correlations between fecal microbiota and dietary intake frequencies. Colored heatmap of Spearman’s rank correlation coefficients for fecal microbiota and dietary intake frequencies. Colors refer to the correlation coefficient direction and magnitude, ranging from − 1 (blue) to 1 (red). * *p* < 0.05 (2-tailed). ** *p* < 0.01 (2-tailed)

In the correlation analysis between gut microbiota and dietary intake (Fig. 6), the abundance of *Bacteroides caccae* was negatively correlated with the daily intake of saturated fat and sodium. The *Barnesiella* genus showed a negative correlation with the daily total calorie intake and protein percentage. The Enterobacteriaceae family exhibited a positive correlation with the percentage of daily protein intake, while *Bacteroides plebeius* was positively correlated with the daily total calorie intake. The *Enterobacter* genus showed a positive correlation with the daily protein intake percentage, while *Acidaminococcus* showed no significant correlation in the dietary analysis.

Furthermore, in the analysis of correlations between the gut microbiota and food consumption frequencies (Fig. 6), the abundance of *Bacteroides caccae* was negatively correlated with the consumption frequency of staple foods (such as bread, steamed buns, and noodles) and with the use frequency of nut oils and seasonings. The abundance of the Enterobacteriaceae was positively correlated with egg consumption frequency, but negatively correlated with the frequency of consuming braised dishes. The *Acidaminococcus* genus showed a significant negative correlation with the frequency of legume consumption.

## Discussion

In this study, we investigated the gut microbiotic characteristics of elderly patients with hypertension. Study participants were individuals aged 65–80 years who were undergoing geriatric health check-ups, with a final selection of 20 participants in the hypertension group and 21 in the normotensive group. Results showed no significant differences in gender or age between the two groups. However, hypertensive patients had a significantly higher BMI, waist circumference, and systolic BP compared to the normotensive group, which aligns with the clinical characteristics of hypertension. Previous studies also confirmed the correlation between hypertension and obesity [20].

In the blood analysis, hypertensive patients had significantly lower TC and LDL-C levels, while other blood parameters showed no significant differences. This phenomenon may be related to the fact that 75% of the hypertensive participants were undergoing antihypertensive medication treatment. These participants may have also received lifestyle modification recommendations during outpatient visits, including weight loss, dietary adjustments, and exercise, which could have contributed to cholesterol reduction [21]. However, LDL-C levels in both groups remained within the normal range (below 130 mg/dL). Results of nutritional intake showed no significant differences between hypertensive patients and the normotensive group in intake of daily total calories, macronutrients, dietary fiber, vitamins, or minerals.

In this study, we found no significant differences in gut microbiotic α-diversity or β-diversity between hypertensive patients and the normotensive group. Previous studies reported inconsistent results for the fecal microbiotic composition in hypertensive individuals. Sun et al. showed that hypertension and systolic BP were inversely associated with measures of α-diversity, including richness and the Shannon diversity index [14]. Palmu et al. also indicated that the α and β diversities of the taxonomic composition were strongly related to BP indexes in age- and sex-adjusted models [22]. On the other hand, in a Spanish cross-sectional study of 29 untreated hypertensive patients and 32 healthy controls, Chao1 richness, Shannon diversity, and β-diversity did not differ between the groups [23]. A Japanese study that included 97 hypertensive, 162 diabetic, and 96 hyperlipidemic participants likewise reported no differences in richness, Shannon diversity, or β-diversity among the three groups [24]. The inconsistencies in these findings suggest that the overall gut microbiotic diversity is not a key factor influencing hypertension. Instead, changes in relative abundances or the presence of specific bacterial taxa may play more significant roles in BP regulation.

We found significant differences in the relative abundances of six bacterial taxa between hypertensive patients and healthy controls. The relative abundances of *Bacteroides caccae* and the *Barnesiella* genus were lower in hypertensive patients, whereas abundances of the Enterobacteriaceae family, *Bacteroides plebeius*, and the *Enterobacter* and *Acidaminococcus* genera were significantly higher. The gut microbiota is influenced by various factors, including ethnicity, age, dietary patterns, medical history, and medication use [25]. In this study, we specifically recruited elderly participants aged 65–80 years and assessed their dietary intake using 24-h dietary recall and a food frequency questionnaire. Additionally, we excluded individuals with a history of related diseases or prior probiotic use to minimize the potential confounding effects of those factors.

Taxonomically, both *Bacteroides caccae* and *Bacteroides plebeius* belong to the genus *Bacteroides*, but their relative abundances exhibited opposite trends, consistent with previous studies [23]. Since *Bacteroides plebeius* primarily produces acetate, *Bacteroides caccae* mainly generates butyrate [26]. These contrasting SCFAs act on different G-protein-coupled receptors (GPCRs) and may partly explain the opposite abundance patterns we observed.

SCFAs, such as acetate, propionate, and butyrate, can activate GPR41 and GPR43 on the surface of intestinal epithelial cells, promoting the production of chemokines and cytokines involved in protective immune responses and tissue inflammation in mice [27]. Moreover, when propionate binds to the GPR41 receptor in the sympathetic nervous system, it enhances sympathetic activity, thereby increasing BP [28]. GPR41 knockout mice exhibit elevated systolic BP, increased pulse pressure, higher end-diastolic pressure, and aggravated perivascular fibrosis [29, 30]. Acetate and propionate raise blood pressure by stimulating renin release through the renal olfactory receptor 78 (Olfr78). In contrast, a 2–3-fold increase in colonic butyrate lowers BP via the colon–vagus nerve pathway [31]. Taken together, SCFAs may exert opposing effects on BP by activating different receptors, stimulating renin release via Olfr78 in the kidney to raise BP, and inducing vasodilation via GPR41 to lower BP. These contrasting actions highlight their complex role in blood pressure regulation.

Additionally, relative abundances of the Enterobacteriaceae family and the *Enterobacter* genus were significantly higher in hypertensive patients. Previous studies showed that Enterobacteriaceae-derived TMAO can activate the NLR family pyrin domain containing 3 (NLRP3) inflammasome in carotid endothelial cells, leading to endothelial dysfunction and its association with hypertension [32]. This bacterial group is also linked to oxidative stress and inflammatory responses, which may accelerate telomere attrition [33]. Notably, its higher prevalence in elderly populations aligns with the age characteristics of participants in this study.

Finally, this study found that the relative abundance of the *Acidaminococcus* genus was significantly higher in hypertensive patients. Previous research indicated that this bacterial group is associated with propionate production [34], and lower propionate levels were correlated with reduced BP [35]. These findings suggest that *Acidaminococcus* may influence BP regulation through SCFAs.

Regarding correlations between the fecal gut microbiota and clinical factors in this study, the BMI was negatively correlated with *Bacteroides caccae* but positively correlated with the *Enterobacter* and *Acidaminococcus* genera. Previous studies also reported that *Bacteroides caccae* is associated with lower metabolic syndrome scores, fasting blood glucose, and insulin resistance [36]. In contrast, *Enterobacter* was found to be more abundant in obese patients [37], and its endotoxin production may contribute to obesity development. Similarly, *Acidaminococcus* was significantly associated with obesity in Hispanic populations [38], further supporting the correlations observed in this study.

According to results of this study, hemoglobin levels were positively correlated with the *Barnesiella* genus, while WBC counts were negatively correlated with the Enterobacteriaceae family and the *Enterobacter* genus. Previous studies showed that gut dysbiosis was associated with various hematological disorders, including iron-deficiency anemia, thrombosis, thrombocytosis, thrombocytopenia, and hematologic malignancies [37]. However, no studies have yet explored the relationship between the *Barnesiella* genus or the Enterobacteriaceae family and hematological diseases, highlighting a potential direction for future research.

In the present study, the relative abundance of *Bacteroides caccae* declined with increasing daily intakes of saturated fat and sodium. Similar patterns have been observed in two recent experimental models.

In rats, a lard-based high-fat diet markedly amplified the blood pressure increase associated with obstructive sleep apnea, accompanied by a loss of butyrate-producing taxa; this hypertensive phenotype was replicated in naïve rats through fecal transplantation [39]. In humans, a short-term high-sodium diet reduced total *Bacteroides*, shifted the microbial community toward *Prevotella*, and was associated with increased expression of epithelial sodium channel (ENaC) genes that promote intestinal sodium absorption [40]. Taken together with our findings, these reports support the observed negative association between *Bacteroides caccae* and saturated-fat or sodium intake, and suggest possible mechanisms—namely, diminished butyrate production under high-fat conditions and ENaC-mediated sodium retention under high-salt conditions—through which such diets may influence gut microbiota composition and, ultimately, blood pressure regulation.

However, this study has several limitations. First, the small sample size and recruitment of all participants from a single hospital’s health examination cohort may limit the generalizability of the results. Second, approximately 75% of hypertensive participants were receiving antihypertensive medication, which may have influenced the gut microbiotic composition. Third, we did not collect data on potential confounders such as physical activity, socioeconomic status, or recent illness, all of which may affect both gut microbiota and hypertension risk. The absence of these variables limits our ability to fully adjust for confounding effects. Finally, due to the cross-sectional design of the study, we cannot infer causal relationships between gut microbiota and hypertension.

In conclusion, the study indicates that older adults with hypertension had gut microbiota imbalances linked to higher BMI and blood pressure. Certain beneficial bacteria, such as *Bacteroides caccae*, were less abundant and showed negative associations with unhealthy dietary habits, including high intake of saturated fats, sodium and staple foods. This suggests that dietary pattern and nutrient intake may play a key role in shaping the gut microbiota and could be a target for improving blood pressure in older adults.

## Data Availability

No datasets were generated or analysed during the current study.
